# Cholinergic Enhancement of Brain Activation in Mild Cognitive Impairment during Episodic Memory Encoding

**DOI:** 10.3389/fpsyt.2013.00105

**Published:** 2013-09-17

**Authors:** Shannon L. Risacher, Yang Wang, Heather A. Wishart, Laura A. Rabin, Laura A. Flashman, Brenna C. McDonald, John D. West, Robert B. Santulli, Andrew J. Saykin

**Affiliations:** ^1^Department of Radiology and Imaging Sciences, Center for Neuroimaging, Indiana University School of Medicine, Indianapolis, IN, USA; ^2^Indiana Alzheimer Disease Center, Indiana University School of Medicine, Indianapolis, IN, USA; ^3^Geisel School of Medicine, Dartmouth-Hitchcock Medical Center, Lebanon, NH, USA; ^4^Brooklyn College, The Graduate Center of CUNY, Brooklyn, NY, USA

**Keywords:** functional magnetic resonance imaging, mild cognitive impairment, donepezil (Aricept), Alzheimer’s disease, task-related functional connectivity, episodic encoding

## Abstract

**Objective:** To determine the physiological impact of treatment with donepezil (Aricept) on neural circuitry supporting episodic memory encoding in patients with amnestic mild cognitive impairment (MCI) using functional magnetic resonance imaging (fMRI).

**Methods:** Eighteen patients with MCI and 20 age-matched healthy controls (HC) were scanned twice while performing an event-related verbal episodic encoding task. MCI participants were scanned before treatment and after approximately 3 months on donepezil; HC were untreated but rescanned at the same interval. Voxel-level analyses assessed treatment effects on activation profiles in MCI patients relative to retest changes in non-treated HC. Changes in task-related connectivity in medial temporal circuitry were also evaluated, as were associations between brain activation, task-related functional connectivity, task performance, and clinical measures of cognition.

**Results:** At baseline, the MCI group showed reduced activation during encoding relative to HC in the right medial temporal lobe (MTL; hippocampal/parahippocampal) and additional regions, as well as attenuated task-related deactivation, relative to rest, in a medial parietal lobe cluster. After treatment, the MCI group showed normalized MTL activation and improved parietal deactivation. These changes were associated with cognitive performance. After treatment, the MCI group also demonstrated increased task-related functional connectivity from the right MTL cluster seed region to a network of other sites including the basal nucleus/caudate and bilateral frontal lobes. Increased functional connectivity was associated with improved task performance.

**Conclusion:** Pharmacologic enhancement of cholinergic function in amnestic MCI is associated with changes in brain activation and functional connectivity during episodic memory processing which are in turn related to increased cognitive performance. fMRI is a promising biomarker for assessing treatment related changes in brain function.

## Introduction

Alzheimer’s disease (AD) is the most common age-related neurodegenerative disease, affecting millions of older adults worldwide (1). AD is characterized by declining cognitive function, impaired activities of daily living (ADL), and dementia (2). Amnestic mild cognitive impairment (MCI), which features an isolated impairment in memory with relative sparing of other cognitive domains and daily functioning, is considered to be a prodromal stage of AD with an annualized MCI to AD conversion rate of 10–15% (3, 4).

Currently only a few medications are approved for use in patients with AD, although many others are under intensive investigation. The primary FDA-approved therapeutic agents for mild to moderate AD are cholinesterase inhibitors (ChEIs), including donepezil (Aricept), rivastigmine (Exelon), and galantamine (Razadyne, formerly Reminyl). Tacrine (Cognex) the first approved agent of this class has been discontinued. Memantine (Namenda), a glutamatergic NMDA-receptor blocker, is also approved for treatment of moderate to severe AD patients. Effectiveness of these medications in AD has been demonstrated, including mild to moderate improvement of cognition (5), although these effects may be only temporary. However, studies of ChEIs in MCI have shown mixed results at best and currently ChEI treatment is not recommended for MCI (6).

Functional magnetic resonance imaging (fMRI) is a technique which allows for measurement of brain function under a variety of conditions (at rest, during tasks, after drug administration, etc.). Blood oxygen level dependent (BOLD) contrast fMRI assesses relative changes in blood deoxyhemoglobin concentration to provide information on relative activation or deactivation of specific brain regions. Pharmacologic fMRI involves the utilization of BOLD fMRI techniques to assess the effects of pharmacologic agents on regional and global brain activation. This technique has been used in a variety of conditions and with a variety of pharmacologic interventions (7–14).

In patients with MCI and AD, pharmacologic treatment with ChEIs has been evaluated using task-related and resting-state fMRI (10, 15–33). In AD patients, acute dosing with ChEIs resulted in primarily increased activation post-drug treatment during episodic face encoding and recognition, working memory, and visual and attention tasks in task-related regions (15, 19, 22, 29). Chronic dosing of AD patients with ChEIs for varying periods (10–24 weeks) also showed a post-drug effect on brain activation, with patients showing increased activation during episodic encoding and recognition, semantic association, working memory, and visuospatial perceptual tasks in task-related areas (18, 21, 27, 29). Increased activation in task-related regions during episodic encoding after ChEI treatment in AD patients was also associated with improved clinical memory performance (31). Alternatively, a few studies have shown decreased activation in task-related regions after prolonged (5 days) or chronic (12–20 weeks) treatment with ChEIs in AD patients during visual perceptual tasks, such as face matching and location matching (23), episodic encoding and recognition (19), as well as during semantic association and working memory tasks (34). Further, AD patients who did not show improved cognition after chronic dosing with ChEI (“non-responders”) showed mostly decreased activation post-drug in task-related areas during semantic association and working memory tasks, while patients who showed improved cognition after ChEI treatment (“responders”) showed primarily increased activation during these tasks in task-specific regions (25). Finally, resting-state fMRI studies in AD patients treated with chronic ChEIs showed increased functional connectivity in a hippocampal network, a cingulate network, and the default mode network (DMN) post-drug treatment (28, 30, 33).

Only a few studies have evaluated ChEI treatment effects on fMRI brain activation in MCI patients. Acute dosing of MCI patients with ChEIs results in increased activation post-drug in task-specific regions during episodic encoding and recognition (19), while prolonged treatment (5–7 days) resulted in increased activation post-drug during episodic encoding, working memory, and spatial navigation tasks (16, 20). When comparing AD and MCI patients after acute ChEI dosing, MCI patients demonstrated increased activation during face recognition after treatment, while AD patients showed increased activation during face encoding (19). On the other hand, one study demonstrated decreased activation during episodic encoding and recognition in task-related regions after more prolonged (5 days) dosing with galantamine in MCI patients (19). Chronic ChEI treatment (11–24 weeks) in MCI patients primarily results in increased activation in task-related regions post-drug during working memory, episodic encoding, and episodic recognition tasks (17, 24, 32). Pa et al. (32) also evaluated functional connectivity during episodic encoding and demonstrated increased connectivity in a fusiform network after chronic treatment with donepezil relative to untreated MCI patients (32).

Although results have been mixed, the administration of ChEIs in patients with MCI and AD generally enhances brain activation in task-dependent regions and brain connectivity measured during resting-state fMRI. However, to date no study has evaluated changes in brain activation and task-related functional connectivity during an auditory verbal episodic memory task in MCI patients after treatment with donepezil, as all previous studies have used visual encoding tasks. Furthermore, no study has evaluated changes in brain activation and connectivity during episodic encoding relative to changes over the same time period in a healthy control (HC) population. The overall goal of the present study was to evaluate the effect of a 3-month treatment period with donepezil (Aricept) on memory circuitry activation pattern and connectivity during episodic memory encoding in MCI patients using event-related fMRI, as well as the relationship between these fMRI measures and cognition before and after treatment. We hypothesized that MCI patients would show increased activation in task-related regions (e.g., hippocampus), as well as greater deactivation in regions of the DMN (e.g., precuneus/posterior cingulate cortex), after donepezil treatment. In addition, we hypothesized that increased activation in task-dependent regions and greater deactivation in the DMN would be associated with better cognitive performance. We also hypothesized that MCI patients would show improved task-related functional connectivity in cholinergic networks associated with the hippocampus and episodic encoding. Finally, we hypothesized that increased functional connectivity in task-related regions would be associated with improved cognition.

## Materials and Methods

### Participants

Participants included 18 patients diagnosed with amnestic MCI and 20 age-matched HC recruited as part of a larger study on brain aging. In the present study, participants were diagnosed as amnestic MCI if they had a combined verbal memory performance on three measures from the California Verbal Learning Test (CVLT) (35, 36) and two measures from the Weschler Memory Scale-III (WMS-III) (37) Logical Memory subtest which fell more than one standard deviation below the performance of the HC group mean, after adjustment for age, education level, and gender. MCI participants were further classified using the recent distinction of early and late MCI [EMCI, LMCI; Ref. (38)] as follows: (1) EMCI patients had a 1–1.49 SD deficit in episodic memory performance; and (2) LMCI patients had a 1.50 SD or greater deficit. In this sample of 18 MCI participants, 2 were classified as EMCI and 16 were classified as LMCI. Further analysis of the difference between these groups demonstrated no significant effect of MCI stage on fMRI activation after donepezil treatment, although the comparison was underpowered due to the small group sizes. Thus, in the present study all MCI participants were combined into a single MCI group. In addition, diagnoses for all participants were reviewed on a case-by-case basis using a multidisciplinary consensus approach and applying the Petersen MCI criteria (2, 3) and the Clinical Dementia Rating (CDR) scale (39). Additional information about participant recruitment, exclusion and inclusion criteria, and study design is available in previous reports (17, 40). All participants provided written informed consent according to the Declaration of Helsinki and all procedures were approved by the Dartmouth College Committee for the Protection of Human Subjects.

### Donepezil protocol

All MCI patients were cholinesterase inhibitor naïve prior to treatment. In addition, we excluded any participants taking other medications that could affect cognition, hemodynamic response in the scanner, and/or cholinergic function. Initially, patients with MCI were provided information on treatment options and all included participants elected to start treatment with donepezil hydrochloride. Participants were carefully monitored by a geriatric psychiatrist (RBS). Participants were started on a 5 mg dose, which was increased to 10 mg after approximately 4 weeks. Reported side effects included mild gastrointestinal symptoms, sleep disturbance, and leg cramps, which are all well-known for this class of medication. No unexpected or serious adverse effects occurred in this study. One participant discontinued donepezil just prior to the second scanning session and initiated treatment with galantamine (Reminyl). All other MCI participants remained on donepezil through the second scan session. At the time of the second scan session, MCI participants were on donepezil for an average of 90.75 (±27.76) days, of which they received 10 mg of donepezil for 54.83 (±22.63) days.

### Clinical and neuropsychological assessments

All participants underwent a comprehensive clinical and neuropsychological battery. Psychometric tests included, but were not limited to: the Mini-Mental State Exam (MMSE) (41, 42), the Mattis Dementia Rating Scale (DRS) (43), the CVLT [sum of trials 1–5 (total), Short Delay Free Recall, and Long Delay Free Recall] (35, 36), and WMS-III Logical Memory Immediate and Delayed Recall (37). In addition, both the participant and an informant were given measures to assess cognitive complaints, which are detailed in a previous report (40). These include a Memory Assessment Questionnaire (40), which is adapted in part from the Functional Activities Questionnaire (44), the Memory Self-Rating Questionnaire (45), the self and informant versions of the Neurobehavioral Function and ADL Rating Scale (46), self and informant versions of the Questionnaire on Cognitive Decline in the Elderly (47), four cognitive items from the Geriatric Depression Scale (GDS) (48), and 10 cognitive items from a telephone-based screening for MCI (49). A cognitive complaint index (CCI) was calculated as the percentage of all items endorsed as a complaint for each participant and his or her informant independently, as well as across all metrics (self and informant) (40). Finally, depressive symptoms were assessed using the GDS. Since the MCI patient population is known to have complaints about memory, we utilized an adjusted GDS score to measure depression, which subtracts from the total score the endorsement of items pertaining to cognitive difficulty, if applicable.

### fMRI scanning protocols and pre-processing

Healthy control and MCI participants were scanned twice, with an average interval between scans of 111.8 (±30.0) days. All scans were completed on a GE Signa 1.5 T Horizon LX scanner with echo speed gradients using a standard RF coil. Specific fMRI parameters were as follows: TR = 2500 ms, TE = 40 ms, FOV = 24 cm, NEX = 1. This scanning protocol resulted in 29 contiguous 5 mm sagittal slices in a 64 × 64 matrix with an in-plane resolution of 3.75 mm^2^.

**Figure 1:**
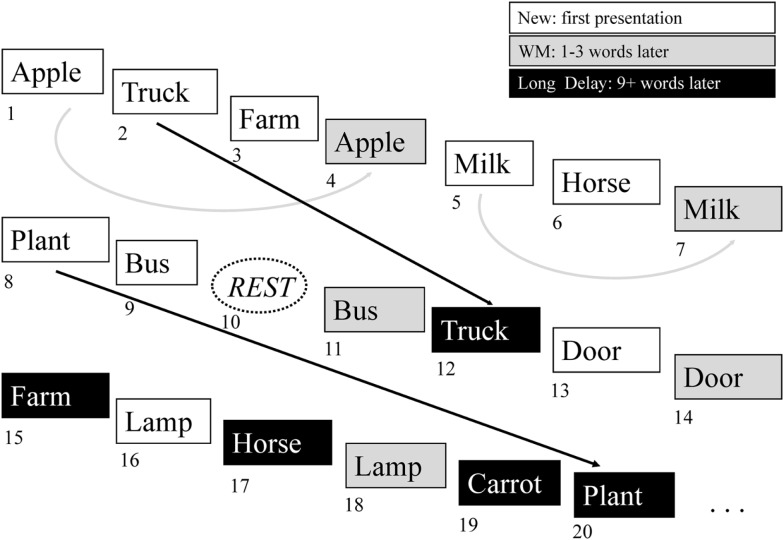
**Continuous verbal episodic memory task**. The episodic memory task used in the present study features continuous encoding and recognition of simple words. The participant responds to each item by indicating whether it is a new item (not previously heard) or an old item (previously heard). The new items represent episodic encoding, as the participant must encode these items for possible future presentation. The old items represent episodic recognition and are divided into working memory items (WM), where the item was presented one to three words prior to the recognition episode, and long delay memory items, where the target item was presented nine or more words prior to the recognition item event. See the text for further description of this task.

The administered task was an auditory event-related continuous performance episodic recognition memory task (12, 50, 51). This task design was adapted from an ERP paradigm that had differentiated medial temporal and frontal contributions to episodic memory (52) and consists of a list of words with a pseudorandom jittered interstimulus interval of 5–8 s and a total task time of 330 s. Each word is presented twice, with the second presentation either immediately following the first (within three words, “working memory” recognition condition), or nine or more items later (“long delay” recognition condition) (Figure 1). The participant responds by button press to each presented word, indicating whether the stimulus is being presented for the first time (“new”) or the second time (“old”). Epochs of rest, in which no word is presented, are also present in the task sequence. Analysis is performed by dividing data into four event types: “rest” (no word presentation), “new” (which is considered an encoding condition), “working memory,” and “long delay”. The “working memory” and “long delay” conditions are also concatenated into a single “old” condition. Contrasts are then made to compare hemodynamic response functions for each of these conditions. In order to evaluate episodic encoding, the contrasts evaluated in the present analysis include: “new” greater than “old” (NEWgtOLD) and “new” greater than “rest” (NEWgtREST). Response accuracy and reaction time for each condition were also evaluated. Prior to the scans, participants rehearsed alternate version of the task outside the scanner to ensure understanding of the instructions and task requirements. All participants demonstrated sufficient task comprehension before scanning.

After quality control to rule out scanning artifacts and/or excessive motion, all fMRI data were pre-processed using standard procedures in the Statistical Parametric Mapping package (SPM5)^1^. Briefly, scans were spatially aligned to remove any motion-related signal change, normalized to a standard space atlas [Montreal Neurologic Institute (MNI)], resampled to 2 mm^3^ isotropic voxels, and smoothed to a full width at half maximum of 8 mm. Two runs of equivalent alternate forms of the task were completed for each participant at each scanning session and the both the fMRI and performance results were averaged across the two runs for further analysis. Two participants failed quality control for one of the two task trials and thus, only one trial was used for these two participants in analyses. Contrast images were created using random effects analyses for the target comparisons (NEWgtOLD, NEWgtREST) and analyzed as described below.

### Task-related region of interest connectivity analysis

Task-related functional connectivity was assessed using an updated version of previously published psychophysiological interaction (PPI) analysis methods (53). This approach evaluates how regional network activation covaries in relation to a target region of interest (ROI) during the episodic memory task (54–56). A seed region was delineated as a significant cluster within the right medial temporal lobe that was identified in the group-by-time comparisons of fMRI activation from the primary contrast to detect episodic encoding activation (NEWgtOLD). First, the drift effect was extracted from the time series using AFNI 3dSynthesize^2^ and then the temporal trend was removed. After that, averaged time series of the seed region were deconvolved with a gamma variant model to generate the first regressor for the PPI analysis (54). A general linear model was then constructed for each participant using three regressors: (1) the physiological variable, represented by the deconvolved bold signal from the seed region, (2) the psychological variable, represented by the task condition type (encoding, coded 1 for new events and 0 for old events) and (3) the interaction term between the first and the second regressor. Contrasts for this interaction term revealed brain regions considered to covary as a functional network with the seed region for each participant (54, 55).

### Statistical analyses

**Figure 2:**
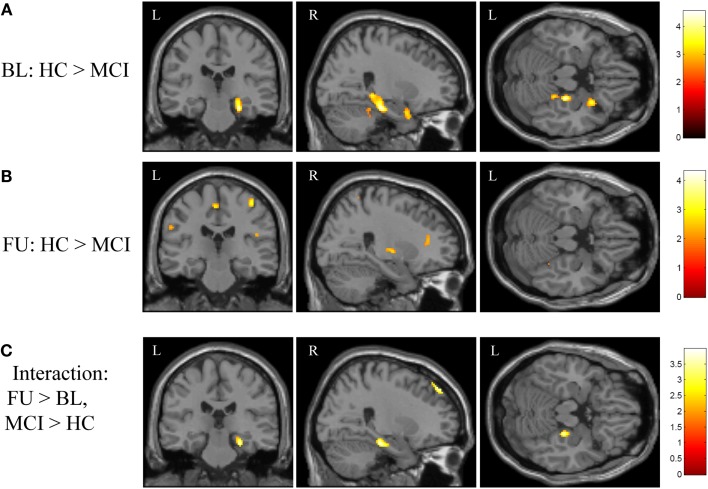
**Effects of diagnostic group and time on brain activation during episodic encoding relative to episodic recognition**. **(A)** HC participants (*n* = 20) showed significantly greater activation than MCI patients (*n* = 18) at baseline in the right medial temporal lobe (right hippocampus and parahippocampal gyrus), along with other cortical and subcortical regions. **(B)** No difference between MCI and HC participants was seen in the medial temporal lobe after MCI participants underwent approximately 3 months of donepezil treatment, although a few other regions remained significant. **(C)** The interaction of diagnostic group-by-time demonstrated that MCI patients showed a greater increase in brain activation in the right medial temporal lobe after donepezil treatment relative to HC. All panels are displayed at a voxel-wise threshold of *p* < 0.01 (uncorrected) and a minimum cluster size (*k*) = 50 voxels. However, most of the observed clusters have local maxima that reach a voxel-wise threshold of *p* < 0.001 (uncorrected; see Table A1 in Appendix). See the Section “Results” and Table A1 in Appendix for additional results. FU = follow-up, BL = baseline, MCI = mild cognitive impairment, HC = healthy older adults, R = right, L = left.

Contrast images were compared between diagnostic groups (HC and MCI) on a voxel-by-voxel basis in SPM8. We used SPM8 due to the ease of navigating results. However, the same analysis in SPM5 generated nearly identical results. We first compared the effect of diagnostic group (HC vs. MCI) at each of the two timepoints (baseline and follow-up). In addition, since we were interested in the effects of donepezil on brain activation in the MCI group, an interaction between group and time (baseline and follow-up) was evaluated. All analyses used a whole-brain mask and were displayed at a voxel-wise threshold of *p* < 0.01 (uncorrected) and minimum cluster size (*k*) = 50 voxels. However, note that in all reported clusters many of the local maxima met a voxel-wise significance threshold of *p* < 0.001 (uncorrected; see Tables A1 and A2 in Appendix). In summary, for each contrast condition (NEWgtOLD, NEWgtREST), the following comparisons were evaluated: two diagnosis group-by-time interactions (follow-up > baseline for MCI > HC and for HC > MCI) and four cross-sectional analyses (HC > MCI at baseline, HC > MCI at follow-up, MCI > HC at baseline, MCI > HC at follow-up). In the present results, we present results from selected analyses which demonstrated significant and biologically relevant effects. Significant voxels and/or clusters were reported in Tables A1 and A2 in Appendix if they had a *Z*-value ≥3.5 or a cluster-wise significance of *p* < 0.05 (uncorrected) and a *Z*-value ≥3.0 (maximum of three local maxima reported for each significant cluster). Anatomical labels for each significant region were determined by converting the MNI coordinates of the peak voxel to Talairach coordinates and evaluating these coordinates using the Talairach Client (version 2.4)^3^. In addition, ROIs from two clusters of interest [right medial temporal lobe cluster in NEWgtOLD, follow-up > baseline, MCI > HC (see Figure 2C); medial parietal cluster from NEWgtREST, MCI > HC at baseline (see Figure 4A)] were extracted for each participant’s contrast images (NEWgtOLD, NEWgtREST, respectively) using MarsBaR (57). Data were also extracted from each set of contrast images using anatomical ROIs from the bilateral precentral and postcentral gyri as control regions.

Task-related functional connectivity contrast maps were assessed for the effect of diagnostic group and time using a two-way general linear model in SPM8. Briefly, the effects of diagnostic group and time were assessed, generating two interaction contrasts (follow-up > baseline, MCI > HC, and HC > MCI). A whole-brain mask was applied and a voxel-wise threshold of *p* < 0.01 (uncorrected) and *k* = 100 voxels was considered significant. Note that all reported clusters contained multiple local maxima that met a voxel-wise significance threshold of *p* < 0.001 (uncorrected; see Table A3 in Appendix). Significant voxels and/or clusters were reported in Table A3 in Appendix if they had a cluster-wise significance of *p* < 0.05 (uncorrected) and *Z*-value ≥3.0 (maximum of three local maxima reported for each significant cluster). ROIs were then generated from selected significant clusters identified in the diagnostic group-by-time interaction analysis, including from the left basal nucleus/caudate, left medial temporal lobe, and the left frontal lobe. Mean task-related functional connectivity values in the target ROIs were extracted from the contrast maps using MarsBaR (57).

Demographics, *APOE* genotype, family history of dementia, baseline neuropsychological performance, and baseline task performance were compared between diagnostic groups (HC vs. MCI) using an ANCOVA model in SPSS (version 20). Baseline and follow-up fMRI activation values for target contrasts (NEWgtOLD, NEWgtREST), as well task-related functional connectivity measures from the extracted target ROIs were also compared between diagnostic groups. Age, gender, and education were included as covariates where appropriate (i.e., ROI values, neuropsychological, and task performance variables). Change from baseline to follow-up in neuropsychological performance and task performance was computed as the follow-up value minus the baseline value. These change values were compared between groups using an ANCOVA model, covaried for baseline age, gender, and education. Repeated measures models were evaluated to look at the interaction of diagnostic group-by-time on all target ROI measures covaried for age, gender, and education.

The relationships between baseline and change in neuropsychological performance, task performance, and extracted ROI values were assessed using a partial Pearson correlation model, covaried for age, gender, and education (where appropriate), and a Spearman correlation model within the MCI group only. Specifically, associations between baseline and change in neuropsychological and task performance and target ROI values of fMRI activation and connectivity at baseline, follow-up, and the change from baseline to follow-up were evaluated. Only the associations that were found to be significant using both the Pearson and Spearman correlation models are presented. However, only the statistical results for the Pearson correlations are reported.

## Results

### Demographics, neuropsychological, and task performance

No significant differences were observed between MCI and HC participants in age, gender, years of education, *APOE* ϵ4 genotype carrier status, family history of dementia, or depressive symptoms (Table 1). As expected, MCI and HC participants differed on all clinically administered neuropsychological measures at baseline, with MCI participants showing more impaired cognition than HC (Table 1; *p* < 0.001). However, fMRI task performance (accuracy and reaction time) did not differ between MCI and HC participants at baseline. Total, self, and informant cognitive complaints were higher in the MCI patients than in HCs (Table 1; *p* < 0.001). Between baseline and follow-up, MCI participants demonstrated a significant improvement in CVLT total score relative to the change in HC participants (Table 1; *p* < 0.001). In addition, MCI participants had a greater decline in total and self cognitive complaints after treatment (Table 1; *p* = 0.007 and 0.005, respectively). No other significant differences between MCI and HC in psychometric test performance change over the follow-up period were observed. Change in task performance accuracy on long delay items was significantly different between HC and MCI, with MCI participants showing a mild accuracy decline and HC participants showing a marked improvement (Table 1; *p* < 0.05). No other changes in task performance measures were significantly different between HC and MCI participants.

**Table 1:** **Demographic information and neuropsychological performance**.

	HC (*n* = 20)	MCI (*n* = 18)	*p*-Value
Age (years)	71.4 (4.7)	72.3 (6.3)	ns
Education (years)	17.1 (2.4)	16.3 (2.9)	ns
Gender (M, F)	8, 12	10, 8	ns
*APOE* ε4 positive (%)^a^	45.0	50.0	ns
Family history of dementia (% positive)	55.0	55.6	ns
Baseline average memory *Z*-score^b^	0.1 (0.6)	−2.4 (0.7)	<0.001
Baseline CDR – sum of boxes^c^	0.2 (0.2)	1.6 (0.8)	<0.001
Baseline MMSE total score^c^	29.1 (0.9)	26.6 (2.8)	<0.001
Baseline DRS total score^c^	140.8 (2.0)	136.7 (4.0)	<0.001
Baseline CVLT total immediate score^c^	49.3 (7.8)	30.1 (5.9)	<0.001
Baseline CVLT short delay recall score^c^	11.6 (2.1)	4.9 (2.1)	<0.001
Baseline CVLT long delay recall score^c^	12.0 (2.0)	5.4 (2.2)	<0.001
Baseline Logical Memory immediate recall^c^	49.1 (6.9)	33.4 (8.7)	<0.001
Baseline Logical Memory delayed recall^c^	32.4 (5.6)	19.0 (7.6)	<0.001
Baseline GDS (adjusted)^c^	1.0 (1.6)	1.7 (1.9)	ns
Baseline overall CCI^d^^,^^e^	9.0 (4.7)	35.0 (14.2)	<0.001
Baseline self CCI^d^^,^^e^	13.0 (6.5)	43.4 (20.8)	<0.001
Baseline informant CCI^d^^,^^e^	3.8 (4.6)	26.5 (18.5)	<0.001
Baseline task accuracy – new items^c^	96.7 (4.8)	93.9 (9.5)	ns
Baseline task accuracy – WM items^c^	87.9 (17.7)	92.3 (7.1)	ns
Baseline task accuracy – long delay items^c^	62.1 (21.7)	56.9 (22.3)	ns
Baseline task accuracy – overall^c^	86.0 (8.1)	84.3 (7.1)	ns
Baseline task reaction time – new items^c^ (ms)	654 (559)	730 (520)	ns
Baseline task reaction time – old items^c^ (ms)	764 (514)	854 (471)	ns
Change in MMSE total score^c^	0.2 (1.3)	0.4 (1.5)	ns
Change in DRS total score^c^	0.9 (2.2)	1.5 (3.5)	ns
Change in CVLT total immediate score^c^	−1.4 (6.0)	6.1 (5.3)	<0.001
Change in CVLT short delay score^c^	−0.6 (2.7)	0.7 (3.0)	ns
Change in CVLT long delay score^c^	−0.1 (2.4)	0.8 (3.4)	ns
Change in overall CCI^d^^,^^e^	−3.8 (4.7)	−12.3 (10.7)	0.003
Change in self CCI^d^^,^^e^	−5.3 (6.2)	−16.8 (14.6)	0.003
Change in informant CCI^d^^,^^e^	−1.8 (4.3)	−8.5 (12.0)	0.028
Change in task accuracy – new items^c^	−1.1 (5.4)	2.0 (8.5)	ns
Change in task accuracy – WM items^c^	6.6 (18.0)	0.5 (7.7)	ns
Change in task accuracy – long delay items^c^	9.0 (18.3)	−1.7 (23.0)	0.038
Change in task accuracy – overall^c^	3.2 (8.3)	0.7 (5.9)	ns
Change in task reaction time – new items^c^ (ms)	−89 (762)	−154 (678)	ns
Change in task reaction time – old items^c^ (ms)	−175 (592)	−178 (541)	ns

^a^Two MCI participants missing APOE genotype information.

^b^Average memory Z-score is computed as follows: z-scores are calculated using the mean and standard deviation, adjusted for age, gender, and education, from whole study healthy control group for the CVLT total immediate score, CVLT short delay recall score, CVLT long delay recall score, Logical Memory immediate recall, and Logical Memory delayed recall; the average of these five z-scores represents the average memory Z-score.

^c^Raw values are presented but statistical tests include age, gender, and education as covariates.

^d^One HC participant missing all CCI data (total, self, and informant).

^e^Raw values are presented; statistical tests do not include covariates.

HC, healthy control; MCI, mild cognitive impairment; M, male; F, female; ns, not significant; CDR, Clinical Dementia Rating scale; MMSE, Mini-Mental State Exam; DRS, Mattis Dementia Rating Scale – version 2; CVLT, California Verbal Learning Test – version 2; GDS, Geratric Depression Scale; CCI, Cognitive Complaint Index; WM, Working Memory; ms, milliseconds.

### Voxel-wise comparisons

The initial voxel-wise analysis considered the effect of diagnostic group at baseline and follow-up independently. At baseline, HC participants showed significantly more activation during encoding (NEWgtOLD) than MCI patients in the right medial temporal lobe (right hippocampus and parahippocampal gyrus), as well as in the right cerebellum, right uncus, left superior frontal gyrus, and the right superior temporal gyrus (Figure 2A; Table A1A in Appendix). At follow-up, this difference between HC and MCI participants in the right medial temporal lobe was not observed. However, HC participants demonstrated greater activation than MCI in a number of other regions at follow-up that were not different between the diagnostic groups at baseline, including in the brainstem (medulla), right insula, bilateral precentral gyrus, bilateral inferior parietal lobules, right caudate, left medial frontal gyrus, and right putamen (Figure 2B; Table A1A in Appendix). A continued difference between diagnostic groups was also observed in the right cerebellum with HC participants showing greater activation than MCI patients. We then performed a voxel-wise analysis to assess the effects of the interaction of diagnostic group-by-time on activation during encoding (NEWgtOLD) and showed a number of significant clusters. At follow-up relative to baseline, patients with MCI showed a greater increase in activation in the right medial temporal lobe (right hippocampus and parahippocampal gyrus) and right middle frontal gyrus than HC participants (Figure 2C; Table A1B in Appendix).

**Figure 3:**
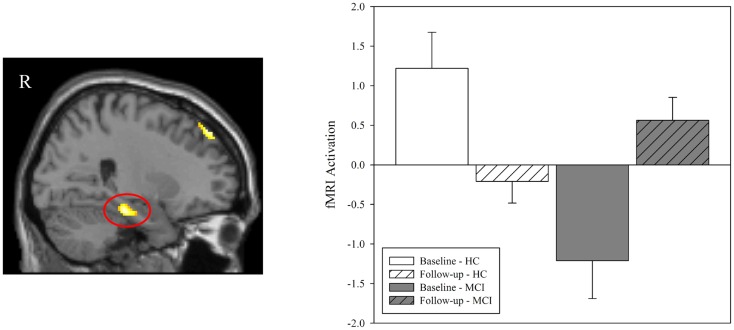
**Effect of diagnostic group and donepezil treatment on medial temporal activation during episodic encoding relative to episodic recognition**. Values for fMRI activation were extracted from a region of interest (ROI) in the right medial temporal lobe (right hippocampus and parahippocampal gyrus) that was observed to be significant in the voxel-wise analysis of the interaction of diagnostic group-by-time (left panel; also see Figure 2C). Group means demonstrated significant effects of diagnostic group and time. Similar to the results seen in the voxel-wise analysis (Figures 2A,B), MCI patients (*n* = 18) showed less activation in the right medial temporal lobe than HC at baseline (*n* = 20; *p* < 0.001) but no significant difference between groups was observed at follow-up (*p* > 0.05). A significant interaction of diagnostic group-by-time was also observed (*p* < 0.001), with MCI patients showing more activation in the right medial temporal lobe after donepezil treatment, as would be expected given the results of the voxel-wise analysis (Figure 2C). MCI = mild cognitive impairment, HC = healthy older adults, ROI = region of interest, R = right, L = left.

In order to further explore the fMRI activation in the right medial temporal lobe cluster that was observed in the voxel-wise diagnostic group-by-time interaction, we generated a ROI from the cluster identified in the interaction analysis of NEWgtOLD (Figure 2C). The mean fMRI activation in this region for each group at baseline and follow-up is shown in Figure 3. As expected considering the voxel-wise results (Figures 2A,B), a significant effect of diagnostic group on fMRI activation is observed at baseline in the right medial temporal lobe (Figure 3; *p* < 0.001) but not at the follow-up scan. Furthermore, a similar significant interaction of diagnostic group-by-time was observed in the right medial temporal lobe ROI (Figure 3; *p* < 0.001) as was observed in the voxel-wise analysis (Figure 2C). Additional analyses using data extracted from “control” regions with fewer cholinergic innervations, such as the primary motor and sensory cortices, showed no significant effects of diagnostic group or treatment on activation at any timepoint (data not shown).

**Figure 4:**
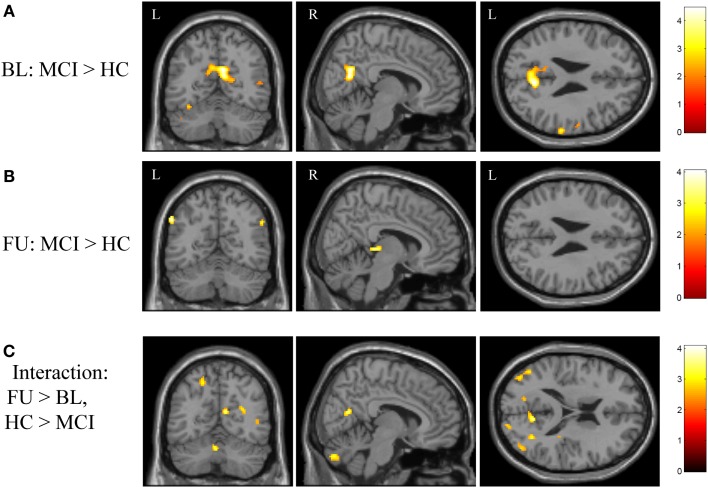
**Effects of diagnostic group and time on brain activation during episodic encoding relative to rest**. **(A)** MCI participants (*n* = 18) showed significantly greater activation than HC participants (*n* = 20) in the medial parietal lobe (precuneus / posterior cingulate) at baseline, as well as in other cortical and subcortical regions. **(B)** However, no difference between MCI and HC participants was seen in the medial parietal region after donepezil treatment in the MCI participants. **(C)** Significant clusters were also observed in the interaction of diagnostic group-by-time analysis. All panels are displayed at a voxel-wise threshold of *p* < 0.01 (uncorrected) and a minimum cluster size (*k*) = 50 voxels. However, all observed clusters include local maxima that meet a voxel-wise threshold of *p* < 0.001 (uncorrected; see Table A2 in Appendix). Further, the medial parietal lobe cluster observed in **(A)** is significant at a cluster-wise threshold of *p* < 0.05 (family-wise error correction for multiple comparisons). See the “Results” section and Table A2 in Appendix for additional results. FU = follow-up, BL = baseline, MCI = mild cognitive impairment, HC = healthy older adults, R = right, L = left.

In addition to considering the contrast of episodic encoding relative to recognition (NEWgtOLD), we evaluated the effect of diagnostic group and time on episodic encoding relative to rest (NEWgtREST). This comparison employing a lower-level baseline may detect regions that are differentially deactivated by the encoding task (i.e., those more active at rest than during the task). We first evaluated the effects of diagnostic group on activation during encoding (NEWgtREST) at baseline and follow-up and a few interesting findings were noted. MCI participants showed significantly greater “activation” at baseline than HC participants in a significant cluster in the bilateral medial parietal lobe [precuneus/posterior cingulate; cluster-wise significant at *p* < 0.05 (family-wise error (FWE) correction for multiple comparisons)], as well as in other significant clusters in the left subthalamic nucleus, left lentiform nucleus/globus pallidus, and right superior parietal lobule (Figure 4A; Table A2A in Appendix). At the follow-up scan, the medial parietal lobe region was no longer different between MCI and HC participants. However, other regions that were more activated in MCI than HC participants at the follow-up scan but showed no difference between the diagnostic groups at baseline included the left inferior parietal lobule, right cerebellum, right thalamus, and right middle frontal gyrus (Figure 4B; Table A2A in Appendix). In the interaction analysis, HC participants showed greater activation than MCI participants at follow-up relative to baseline in the right postcentral gyrus, bilateral cerebellum, right putamen, right insula, right middle temporal gyrus, and the right parietal lobe (Figure 4C; Table A2B in Appendix).

**Figure 5:**
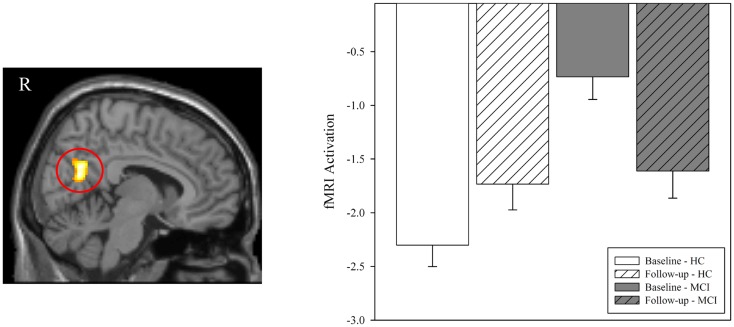
**Effect of diagnostic group and time on brain activation in the medial parietal lobe during episodic encoding relative to rest**. Mean fMRI activation was extracted from a region of interest (ROI) in the medial parietal lobe that was observed to be significantly associated with diagnostic group at baseline (left panel; also see Figure 4A). Group means demonstrated significant effects of diagnostic group and time. Clarifying the results seen in the voxel-wise analysis (Figure 4A), MCI patients (*n* = 18) showed less deactivation rather than more activation in the medial parietal lobe relative to healthy controls at baseline (HC, *n* = 20; *p* < 0.001). However, no significant difference between diagnostic groups was observed at follow-up (similar to results in Figure 4B; *p* > 0.05). A significant interaction of diagnostic group-by-time was also observed (*p* < 0.001), with MCI participants showing more deactivation in the medial parietal lobe after donepezil treatment. MCI = mild cognitive impairment, HC = healthy older adults, ROI = region of interest, R = right, L = left.

In order to further evaluate the medial parietal cluster identified in the cross-sectional analysis as showing greater “activation” at baseline in MCI patients relative to HC, we generated a ROI from the medial parietal cluster (precuneus/posterior cingulate) identified in the baseline comparison of diagnostic groups on the NEWgtREST contrast (Figure 4A). The mean activations in this region for both groups at baseline and follow-up are shown in Figure 5. Rather than greater MCI activation, the results show that MCI patients are deactivating significantly less than HC participants at baseline (Figure 5; *p* < 0.001), but the MCI and HC medial parietal activations are similar at the follow-up scan (Figure 5; *p* > 0.05). Thus, a significant interaction of diagnostic group-by-time was observed, with MCI participants showing greater medial parietal deactivation after donepezil treatment (Figure 5; *p* < 0.001), as would be expected given the voxel-wise findings.

### Associations between cognitive performance and fMRI activations

**Figure 6:**
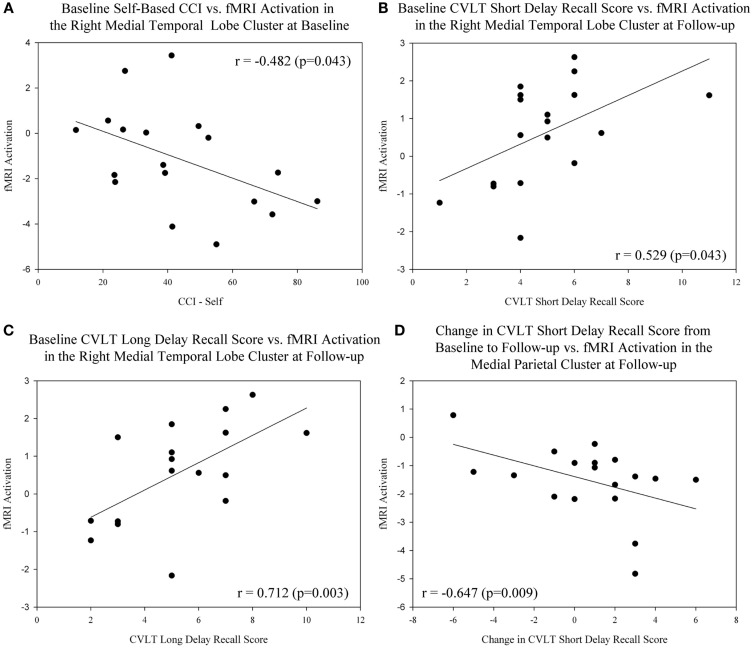
**Association between cognitive complaints and performance and brain activation in target ROIs before and after donepezil treatment in MCI**. **(A)** A significant negative association between the baseline level of self-based cognitive complaints (CCI-Self), and fMRI activation in the right medial temporal lobe at baseline was observed in MCI participants (*n* = 18; *r* = −0.482, *p* = 0.043). Significant associations between baseline memory performance on the CVLT Short Delay Recall [**(B)**; *r* = 0.529, *p* = 0.043] and CVLT Long Delay Recall [**(C)**; *r* = 0.712, *p* = 0.003] and fMRI activation in the medial temporal lobe at the post-drug follow-up scan were also observed. **(D)** Greater improvement in memory performance on the CVLT Short Delay Recall was associated with more deactivation in the medial parietal cluster at the post-drug follow-up scan (*r* = −0.647, *p* = 0.009). Note that all medial temporal lobe values are extracted from NEWgtOLD contrasts and all medial parietal lobe values are extracted from NEWgtREST contrasts. Data are displayed as raw values; however, all statistical results include age, gender, and education as covariates. For additional information, see the Sections “Materials and Methods” and “Results” of the manuscript. NEWgtOLD = new items contrasted with old items (encoding > recognition), NEWgtREST = new items contrasted with rest items (encoding > rest), ROI = region of interest, MCI = mild cognitive impairment, HC = healthy older adults, CVLT = California Verbal Learning Test, CCI = cognitive complaint index.

We evaluated the relationship between baseline, follow-up, and change in measures of cognitive performance and fMRI activation in the target regions of interest (right medial temporal lobe cluster, medial parietal cluster) in MCI participants only. A significant negative association between the baseline level of cognitive complaints from the MCI participant (measured using the CCI) and fMRI activation in the right medial temporal lobe at the baseline scan was observed (Figure 6A; *r* = −0.482, *p* = 0.043). In addition, memory performance at baseline on the CVLT short delay recall score (Figure 6B; *r* = 0.529, *p* = 0.043) and CVLT long delay recall score (Figure 6C; *r* = 0.712, *p* = 0.003) demonstrated a significant positive association with fMRI activation in the right medial temporal lobe at the post-drug follow-up scan. Finally, greater improvement in memory performance on the CVLT short delay recall score was associated with more deactivation in the medial parietal cluster at the post-drug follow-up scan (Figure 6D; *r* = −0.647, *p* = 0.009).

### Task-related functional connectivity

**Figure 7:**
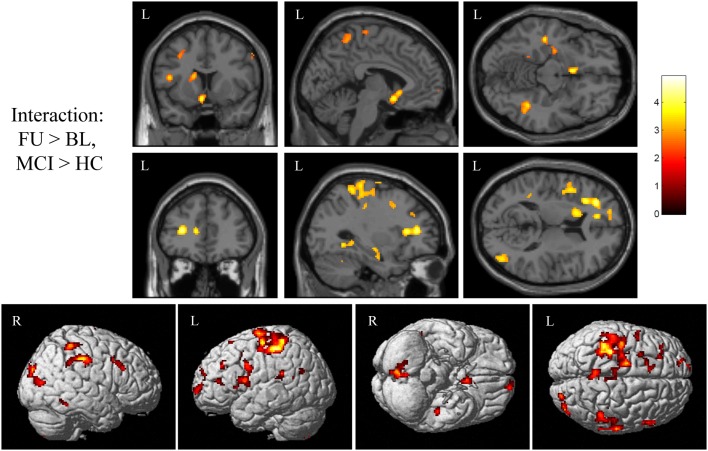
**Interaction of diagnostic group and time on task-related functional connectivity during episodic encoding relative to episodic recall**. MCI patients (*n* = 18) showed a greater increase in task-related functional connectivity in the left basal nucleus/caudate (top row), left medial temporal lobe (top row), left frontal lobe (second row), as well as regions of the precentral and postcentral gyri, parietal lobe, cingulate, lateral temporal lobe, occipital lobe, and cerebellum relative to HC participants (*n* = 20). All panels are displayed at a voxel-wise threshold of *p* < 0.01 (uncorrected) and a minimum cluster size (*k*) = 100 voxels. However, all clusters contain local maxima that reach a voxel-wise threshold of *p* < 0.001 (uncorrected; see Table A3 in Appendix). Further, numerous clusters reach a cluster-wise significance of *p* < 0.05 (family-wise error correction for multiple comparisons), including clusters in the left frontal lobe, basal nucleus/caudate, left parahippocampal gyrus, bilateral postcentral gyrus, left precentral gyrus, right parietal lobe, anterior cingulate, left insula, left temporal lobe, and cerebellum. See the Section “Results” and Table A3 in Appendix for additional results. MCI = mild cognitive impairment, HC = healthy older adults, R = right, L = left.

Next, we sought to evaluate the effect of treatment with donepezil on task-related functional connectivity of the right medial temporal lobe region identified as significant in the interaction analyses of fMRI activation associated with episodic encoding greater than recognition (Figure 2C). Voxel-wise analysis of the diagnostic group-by-time interaction demonstrated a number of significant clusters showing increased connectivity after donepezil treatment in MCI relative to HC, including in the left superior, inferior, and medial frontal gyri, right inferior parietal lobule, bilateral middle frontal gyri, bilateral parahippocampal gyrus, left basal nucleus/caudate, right posterior cingulate, bilateral postcentral gyri, left precentral gyrus, left anterior cingulate, left superior temporal gyrus, left claustrum, right superior parietal gyrus, left paracentral lobule, left insula, right superior occipital gyrus, right middle temporal gyrus, right cuneus, right cingulate, and right cerebellum [Figure 7; multiple clusters significant at a cluster-wise threshold of *p* < 0.05 (FWE), see Table A3 in Appendix].

**Figure 8:**
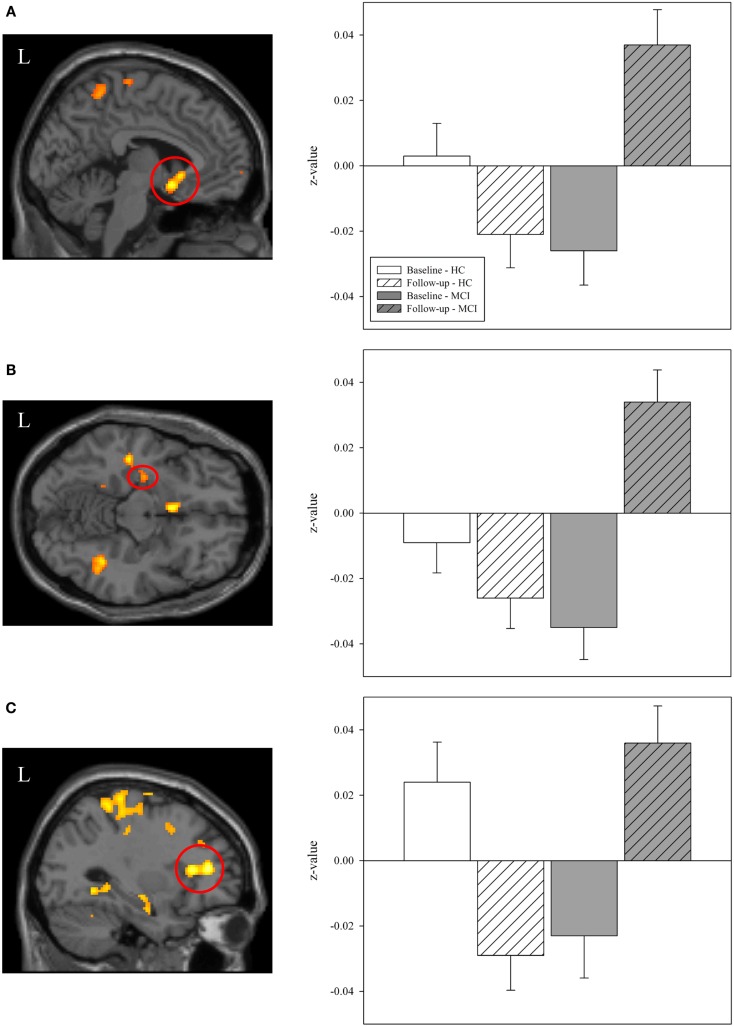
**Effects of diagnostic group and time on task-related functional connectivity in selected regions of interest**. Mean values of task-related functional connectivity were extracted from three regions of interest (ROIs) identified in the interaction analysis of diagnostic group-by-time (left panels; also see Figure 7), including in the **(A)** left basal nucleus/caudate, **(B)** left medial temporal lobe, and **(C)** left frontal lobe. Group means demonstrated significant effects of diagnostic group and time. At baseline, HC participants (*n* = 20) had greater task-related functional connectivity in the left frontal lobe **(C)** than MCI participants. However, at follow-up, MCI patients showed greater task-related functional connectivity than HC participants in all evaluated regions **(A–C)**. As expected, a significant interaction of diagnostic group-by-time was observed for each ROI **(A–C)** with MCI participants showing a greater increase in task-related functional connectivity between the baseline and post-treatment follow-up scans than HC participants (*p* < 0.001). MCI = mild cognitive impairment, HC = healthy older adults, ROI = region of interest, R = right, L = left.

We next extracted selected significant clusters from the task-related functional connectivity analysis of the interaction of diagnostic group-by-time, including significant clusters in the left basal nucleus/caudate, left medial temporal lobe, and left frontal lobe. Our goal was to further explore the distribution of functional connectivity by diagnostic group and time in regions of the cholinergic circuitry. The left basal nucleus/caudate (Figure 8A) and left medial temporal lobe (Figure 8B) clusters showed a significant effect of diagnostic group only at the follow-up scan but not at the baseline scan (for both ROIs, baseline: *p* > 0.05, follow-up: *p* < 0.001). At the follow-up scan, MCI participants showed greater task-related functional connectivity in both target regions than HC participants. However, significant effects of diagnostic group on task-related connectivity in the left frontal region were observed at both the baseline and the follow-up scans (Figure 8C; baseline: *p* = 0.015, follow-up: *p* < 0.001), with HC participants showing greater task-related connectivity at baseline and MCI participants demonstrating greater task-related functional connectivity at follow-up. As would be expected given the voxel-wise analyses, a significant interaction of diagnostic group-by-time was observed for all target ROIs (Figures 8A–C; *p* < 0.001), with HC participants showing no change or a minor change in connectivity but MCI participants showing a significant increase in task-related functional connectivity from baseline to follow-up (i.e., post-donepezil treatment).

### Associations between cognitive and task performance and task-related functional connectivity

**Figure 9:**
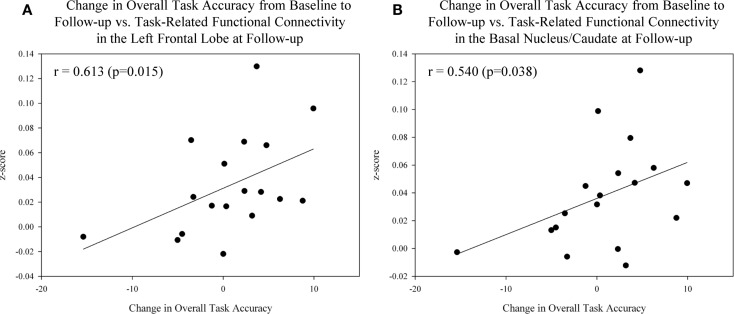
**Association between task performance and task-related functional connectivity in target ROIs after donepezil treatment in MCI patients**. Significant associations between improved overall task accuracy from the baseline to post-drug follow-up scan and task-related functional connectivity in the left frontal lobe [**(A)**; *r* = 0.613, *p* = 0.015] and left basal nucleus/caudate [**(B)**; *r* = 0.540, *p* = 0.038] at the follow-up scan were observed in MCI participants (*n* = 18). See Sections “Materials and Methods” and “Results” of the manuscript for further information. MCI = mild cognitive impairment, HC = healthy older adults, ROI = region of interest.

Lastly, we assessed the association between task-related functional connectivity measures and performance on the episodic memory fMRI task in MCI patients. Significant associations between task-related functional connectivity and measures of overall fMRI task accuracy were observed within the MCI group. Specifically, improved overall task accuracy from the baseline to post-drug follow-up visit was positively associated with increased task-related functional connectivity in the left frontal lobe (Figure 9A; *r* = 0.613, *p* = 0.015) and left basal nucleus/caudate (Figure 9B; *r* = 0.540, *p* = 0.038) at the post-drug follow-up visit.

## Discussion

This study evaluated the effect of donepezil treatment on brain activation in MCI patients during an episodic memory encoding task. Participants with MCI showed increased activation after treatment in the right medial temporal lobe (hippocampus/parahippocampal gyrus), where they failed to activate relative to healthy older adult controls at baseline. The MCI group also showed greater deactivation in the medial parietal lobe after approximately 3 months of treatment with donepezil. Activation in these regions was also associated with pre-drug and post-drug cognitive complaints and performance. A significant increase in task-related functional connectivity between the right medial temporal lobe and the left frontal and medial temporal lobes, as well as bilateral subcortical and cingulate regions, was observed after treatment with donepezil in MCI patients relative to HC. These increases in functional connectivity were also associated with improved fMRI task performance. Overall, MCI patients treated with donepezil appear to show “normalization” of brain activation patterns during episodic encoding in the medial temporal and medial parietal lobes similar to the activation patterns seen in healthy older adults, as well as improved brain connectivity in cholinergic networks.

The increased medial temporal lobe (right hippocampus and parahippocampal gyrus) activation post-treatment observed in MCI patients in the present study is similar to findings in previous studies of both AD and MCI patients. Previous studies evaluating the effect of ChEIs in AD patients have observed increased activation during episodic encoding after treatment in medial and inferior temporal lobe structures, such as the hippocampus, parahippocampal gyri, and fusiform gyri (15, 18). In MCI, two previous studies have also reported increased activation in medial temporal lobe regions during episodic encoding after treatment with ChEI (16, 32). The laterality of observed activation (right > left) in the present study is contrary to the majority of previous studies of episodic encoding in young adults which have primarily implicated the left medial temporal lobe in verbal episodic encoding (58). However, previous studies evaluating activation changes in response to treatment with ChEIs have shown drug effects in both the left and right medial temporal lobes during episodic encoding and recognition (16, 19). Studies of fMRI activation during other cognitive tasks, such as a visual attention task and a location-matching task, have also shown activation changed in both left and right medial temporal lobes in response to ChEI treatment in AD and MCI patients (20, 22, 23, 27). Furthermore, improved cognitive performance has been associated with increased activation in both the left and right medial temporal lobes after treatment with donepezil (31). Resting-state studies have shown changes in the connectivity of a hippocampal network and a cingulate network in the left medial temporal lobe after treatment with ChEIs in patients with AD (28, 30). However, a different study showed changes in connectivity of the DMN in the right medial temporal lobe after donepezil treatment in AD patients (33). Finally, a task-related functional connectivity study showed increased connectivity between the left fusiform and right hippocampus during a face encoding task (32). In sum, the results to date have been mixed regarding the lateralization of ChEI treatment-induced brain activation in MCI and AD. The observed activation changes in the right medial temporal lobe during verbal episodic encoding may represent de-differentiation or compensation in older adults with and without cognitive impairment. A similar pattern of loss of laterality during episodic memory has been reported in the prefrontal cortex in cognitively healthy older adults and patients with prodromal and clinical AD (59). Further studies to investigate the lateralization of medial temporal lobe activation in cognitively healthy and impaired older adult populations during verbal episodic encoding, as well as in the context of treatment with ChEIs, is warranted. Overall, this is the first study to compare change in brain activation during an episodic encoding task in MCI patients after donepezil treatment to healthy older adults scanned with the same fMRI protocol to control for retest effects. The increased activation in the medial temporal lobe seen in the present study, as well as in previous reports, suggests that donepezil may improve brain activation in regions important to memory function in patients with MCI and AD.

The increased deactivation during episodic encoding seen in the medial parietal lobe after treatment with donepezil in MCI patients has not been consistently reported in prior ChEI studies, although a few have reported decreased precuneus/posterior cingulate activation after treatment. Bokde et al. observed a decrease in precuneus activation after drug treatment during a location-matching task in AD patients (23). Goekoop and colleagues also observed a decreased activation after treatment in the left posterior cingulate during an episodic recognition task in MCI but not AD patients (19). We highlight that the present study is the first to demonstrate that the donepezil induced changes in this region reflect appropriately increased deactivation in the DMN rather than reduced task-related activation.

Resting-state connectivity studies in AD patients treated with donepezil have evaluated functional networks which include the medial parietal lobe, such as the DMN and a functional hippocampal network. These studies have shown increased connectivity of the DMN, a hippocampal functional network, and a posterior cingulate functional network post-treatment (28, 30, 33). Improved connectivity in functional or resting-state networks important for memory may underlie the increased deactivation observed in MCI patients. Again, the results suggest that treatment of MCI patients with donepezil may somewhat “normalize” brain activation patterns during episodic encoding, resulting in a pattern of deactivation in the medial parietal lobe and activation in the medial temporal lobe which more closely resembles the activation patterns of healthy older adults.

Previous reports assessing resting-state or task-related functional connectivity have focused solely on AD patients treated with ChEIs. Thus, this is the first study to evaluate task-related functional connectivity before and after treatment with donepezil in MCI. The observed increase in functional connectivity is similar to the previous findings in AD patients (28, 30, 33). The observed significant cluster in the basal nucleus/caudate is particularly interesting, as this cluster is in close proximity to the nucleus basalis of Meynert, which is known to provide cholinergic innervation to the hippocampus and shows early degeneration of cholinergic neurons in AD (60–62). Thus, the increased task-related functional connectivity of the right hippocampus/parahippocampal gyrus with this region may reflect improved function of cholinergic neurons from the nucleus basalis of Meynert to the hippocampus in MCI patients after treatment. Other significant clusters, including in the left medial temporal and frontal lobes, also provide evidence that treatment with donepezil improves the function of cholinergic networks during episodic encoding in MCI patients. The observed associations of increased functional connectivity in target regions and improved cognition further underscores the importance of functional connections of the cholinergic network in memory and cognition. Therefore, not only does donepezil potentially improve local functioning of regions important for episodic encoding (i.e., medial temporal lobe, medial parietal lobe), but treatment of MCI patients with donepezil may also improve the connectivity of cholinergic networks, likely allowing for better task performance and improved cognitive function.

The present study has a few limitations. The absence of a placebo-controlled MCI group impedes our ability to assess the change that may occur in the absence of treatment in MCI patients over time. This group would be an interesting comparison group for both treated MCI patients and HC participants. Future studies including a placebo-controlled MCI group, along with treated MCI and HC participants, would provide additional important information about the effects of ChEI treatment in MCI and aging. Secondly, the relatively short-term nature of the donepezil treatment in the present study (10–12 weeks) may not fully capture any potential longer term beneficial effects of ChEI treatment in MCI. Future research with follow-up over a longer period of time would help to clarify the clinical and functional implications of long-term ChEI treatment. Changes in blood flow and vascular function in response to treatment with ChEIs have been reported in AD and MCI (30, 63–65), as well as in older adults with or without cognitive impairment in the absence of drug treatment. Therefore, any observed changes in brain activation could be related to changes in blood flow or hemodynamic coupling due to aging or donepezil treatment. Specifically, these changes may affect the reliability and stability of the BOLD fMRI signal, which would complicate data interpretation. However, the absence of significant group or drug treatment effects in “control” regions of the brain that are not highly innervated with cholinergic pathways suggests the observed findings cannot only be attributed to changes in blood flow. Future studies utilizing advanced MRI techniques to measure perfusion [i.e., arterial spin labeling (ASL)] may further clarify the relative contribution of vascular changes in ChEI treatment effects. Finally, although the present study is among the largest of its type, the modest sample sizes nonetheless limit statistical power and ability to assess more complex relationships between brain activation, cognitive performance, and genetic variations before and after donepezil treatment. Due to the limited sample size, we chose to use a less stringent threshold for the voxel-wise comparisons and observed activation changes in hypothesized brain regions. However, future studies in expanded cohorts would be beneficial and are needed to replicate the present findings.

In sum, MCI patients treated with donepezil show increased brain activation and deactivation in task-related regions and increased task-related functional connectivity during episodic encoding relative to pre-treatment baseline. Specifically, MCI patients show increased medial temporal lobe activation, increased medial parietal lobe deactivation, and improved task-related connectivity of cholinergic networks after approximately 3 months of cholinergic enhancement with donepezil. On-drug activation patterns and connectivity of functional cholinergic networks in MCI participants are similar to those observed in healthy older adults, suggesting a possible “normalization” of brain activation and connectivity after treatment with donepezil, effects which are associated with memory performance and general cognition.

## Conflict of Interest Statement

Dr. Saykin has received investigator-initiated research funding from Pfizer, Welch Allyn and Siemens Healthcare; has served as a consultant or advisory board member for Siemens Healthcare, Pfizer, and Eli Lilly. All other authors report no conflicts of interest.

## Footnotes

^1^http://www.fil.ion.ucl.ac.uk/spm/

^2^http://afni.nimh.nih.gov/

^3^http://www.talairach.org/client.html

## Appendix

**Table A1:** **Brain activation during episodic memory encoding task (new > old)**.

Contrast	*T*-value	*Z*-value	Voxel*p* (unc.)	Cluster*p* (unc.)	No. of Voxelsin Cluster (*k*)	Cluster *p*(FWE/FDR)	MNI coordinates			Nearest gray matter region
							*X*	*Y*	*Z*	
**(A) EFFECT OF GROUP AT BASELINE AND FOLLOW-UP**										
NEWgtOLD: BL, HC > MCI	4.53	4.21	<0.001	0.003	300	0.25/0.19	26	−24	−20	Right parahippocampal gyrus (BA 35)
NEWgtOLD: BL, HC > MCI	3.53	3.36	<0.001				26	−36	−4	Right hippocampus
NEWgtOLD: BL, HC > MCI	3.16	3.04	0.001				24	−38	−20	Right cerebellum – culmen
NEWgtOLD: BL, HC > MCI	4.10	3.86	<0.001	0.012	210	0.62/0.32	30	8	−24	Right uncus (BA 28)
NEWgtOLD: BL, HC > MCI	3.28	3.14	0.001	0.038	131	0.96/0.53	−6	−4	70	Left superior frontal gyrus (BA 6)
NEWgtOLD: BL, HC > MCI	3.27	3.13	0.001	0.033	141	0.94/0.53	52	2	−4	Right superior temporal gyrus (BA 22)
NEWgtOLD: FU, HC > MCI	4.31	4.03	<0.001	0.161	55	1.00/0.85	4	−34	−58	Brainstem: medulla
NEWgtOLD: FU, HC > MCI	3.95	3.73	<0.001	0.130	65	1.00/0.85	48	−30	20	Right insula (BA 13)
NEWgtOLD: FU, HC > MCI	3.92	3.71	<0.001	0.069	97	1.00/0.85	−52	8	14	Left precentral gyrus (BA 44)
NEWgtOLD: FU, HC > MCI	3.88	3.67	<0.001	0.100	78	1.00/0.85	10	−82	−38	Right cerebellum – pyramis
NEWgtOLD: FU, HC > MCI	3.33	3.19	0.001	0.009	227	0.53/0.74	42	−38	52	Right inferior parietal lobule (BA 40)
NEWgtOLD: FU, HC > MCI	3.31	3.17	0.001				40	−22	60	Right precentral gyrus (BA 4)
NEWgtOLD: FU, HC > MCI	3.29	3.16	0.001	0.033	141	0.94/0.81	38	−46	6	Right caudate tail
NEWgtOLD: FU, HC > MCI	3.21	3.08	0.001	0.016	189	0.73/0.74	−32	−50	44	Left inferior parietal lobule (BA 40)
NEWgtOLD: FU, HC > MCI	3.21	3.08	0.001	0.050	115	0.99/0.81	−2	−28	56	Left medial frontal gyrus (BA 6)
NEWgtOLD: FU, HC > MCI	3.11	2.99	0.001	0.022	165	0.85/0.74	28	−16	4	Right putamen
**(B) INTERACTION OF GROUP X TIME**										
NEWgtOLD: FU > BL, MCI > HC	3.97	3.74	<0.001	0.172	52	1.00/0.88	36	0	64	Right middle frontal gyrus (BA 6)
NEWgtOLD: FU > BL, MCI > HC	3.83	3.63	<0.001	0.1	78	1.00/0.88	26	−26	−18	Right parahippocampal gyrus (BA 35)

BA, Brodmann Area; BL, baseline scan; FU, follow-up scan; HC, healthy control; FWE, family-wise error correction for multiple comparisons; FDR, false discovery rate correction for multiple comparisons; *k*, number of voxels in cluster; MCI, mild cognitive impairment; MNI, Montreal Neurologic Institute; unc., uncorrected for multiple comparisons.

**Table A2:** **Brain activation during episodic memory encoding task (new > rest)**.

Contrast	*T*-value	*Z*-value	Voxel*p* (unc.)	Cluster*p* (unc.)	No. of Voxelsin Cluster (*k*)	Cluster *p*(FWE/FDR)	MNI coordinates			Nearest gray matter region
							*X*	*Y*	*Z*	
**(A) EFFECT OF GROUP AT BASELINE AND FOLLOW-UP**										
NEWgtREST: BL, MCI > HC	4.46	4.15	<0.001	<0.001	683	**0.01/0.01**	8	−60	26	Right cingulate gyrus (BA 31)
NEWgtREST: BL, MCI > HC	3.79	3.59	<0.001				−4	−66	28	Left precuneus (BA 31)
NEWgtREST: BL, MCI > HC	3.20	3.07	0.001				14	−54	18	Right posterior cingulate (BA 31)
NEWgtREST: BL, MCI > HC	4.00	3.77	<0.001	0.054	129	0.98/0.89	−14	−12	−10	Left midbrain – subthalamic nucleus
NEWgtREST: BL, MCI > HC	3.78	3.58	<0.001				−20	−6	−10	Left lentiform nucleus – globus pallidus
NEWgtREST: BL, MCI > HC	3.41	3.26	0.001	0.018	210	0.72/0.82	64	−30	26	Right superior parietal lobule (BA 40)
NEWgtREST: FU, MCI > HC	4.03	3.80	<0.001	0.108	86	1.00/0.89	−56	−62	38	Left inferior parietal lobule (BA 40)
NEWgtREST: FU, MCI > HC	3.74	3.54	<0.001	0.094	94	1.00/0.89	34	−78	−24	Right cerebellum – declive
NEWgtREST: FU, MCI > HC	3.68	3.50	<0.001	0.069	113	0.99/0.89	6	−30	2	Right thalamus – pulvinar
NEWgtREST: FU, MCI > HC	3.57	3.40	<0.001	0.022	193	0.80/0.89	36	12	50	Right middle frontal gyrus (BA 6)
**(B) INTERACTION OF GROUP × TIME**										
NEWgtREST: FU > BL, HC > MCI	4.07	3.83	<0.001	0.021	198	0.78/0.69	54	−28	40	Right postcentral gyrus (BA 2)
NEWgtREST: FU > BL, HC > MCI	3.27	3.13	0.001				64	−20	36	Right postcentral gyrus (BA 3)
NEWgtREST: FU > BL, HC > MCI	3.13	3.01	0.001				46	−30	30	Right postcentral gyrus (BA 2)
NEWgtREST: FU > BL, HC > MCI	3.93	3.71	<0.001	0.047	138	0.97/0.69	−4	−70	−24	Left cerebellum – declive
NEWgtREST: FU > BL, HC > MCI	3.84	3.64	<0.001	0.025	182	0.84/0.69	−6	−76	−38	Left cerebellum – pyramis
NEWgtREST: FU > BL, HC > MCI	3.55	3.38	<0.001				−8	−68	−36	Left cerebellum – pyramis
NEWgtREST: FU > BL, HC > MCI	3.23	3.1	0.001				8	−82	−36	Right cerebellum – pyramis
NEWgtREST: FU > BL, HC > MCI	3.74	3.55	<0.001	0.066	116	0.99/0.69	26	−4	0	Right putamen
NEWgtREST: FU > BL, HC > MCI	3.72	3.53	<0.001	0.146	69	1.00/0.89	30	−56	−34	Right cerebellum – culmen
NEWgtREST: FU > BL, HC > MCI	3.60	3.43	<0.001	0.019	205	0.75/0.69	40	−28	−2	Right insula (BA 13)
NEWgtREST: FU > BL, HC > MCI	3.46	3.31	<0.001	0.042	146	0.95/0.69	48	−70	8	Right middle temporal gyrus (BA 39)
NEWgtREST: FU > BL, HC > MCI	3.14	3.02	0.001	0.035	159	0.92/0.69	24	−42	56	Right parietal sub-gyral (BA 40)
NEWgtREST: FU > BL, HC > MCI	3.11	3.00	0.001	0.050	134	0.97/0.69	30	−58	22	Right middle temporal gyrus (BA 39)
NEWgtREST: FU > BL, MCI > HC	3.69	3.51	<0.001	0.117	81	1.00/0.78	−28	32	44	Left middle frontal gyrus (BA 9)

BOLD, significant at a cluster-wise threshold of p < 0.05 corrected for multiple comparisons (FWE and/or FDR).

BA, Brodmann Area; BL, baseline scan; FU, follow-up scan; HC, healthy control; FWE, family-wise error correction for multiple comparisons; FDR, false discovery rate correction for multiple comparisons; k, number of voxels in cluster; MCI, mild cognitive impairment; MNI, Montreal Neurologic Institute; unc., uncorrected for multiple comparisons.

**Table A3:** **Task-induced functional connectivity during episodic memory encoding (new > old)**.

Contrast	*T*-value	*Z*-value	Voxel*p* (unc.)	Cluster*p* (unc.)	No. of voxelsin cluster (*k*)	Cluster *p*(FWE/FDR)	MNI coordinates			Nearest gray matter region
							*X*	*Y*	*Z*	
**INTERACTION OF GROUP × TIME**										
NEWgtOLD: FU > BL, MCI > HC	4.91	4.71	<0.001	<0.001	1571	<**0.001/** <**0.001**	−18	−10	74	Left superior frontal gyrus (BA 6)
NEWgtOLD: FU > BL, MCI > HC	4.63	4.46	<0.001				−38	−36	54	Left postcentral gyrus (BA 40)
NEWgtOLD: FU > BL, MCI > HC	4.07	3.96	<0.001				−18	−8	54	Left frontal sub-gyral (BA 6)
NEWgtOLD: FU > BL, MCI > HC	4.34	4.20	<0.001	<0.001	339	**0.03/0.008**	68	−24	42	Right postcentral gyrus (BA 1)
NEWgtOLD: FU > BL, MCI > HC	3.10	3.05	0.001				66	−42	36	Right inferior parietal lobule (BA 40)
NEWgtOLD: FU > BL, MCI > HC	3.06	3.00	0.001				52	−26	42	Right postcentral gyrus (BA 2)
NEWgtOLD: FU > BL, MCI > HC	4.22	4.09	<0.001	<0.001	295	**0.064/0.014**	−30	40	12	Left frontal sub-gyral
NEWgtOLD: FU > BL, MCI > HC	3.94	3.84	<0.001				−32	26	10	Left inferior frontal gyrus (BA 45)
NEWgtOLD: FU > BL, MCI > HC	3.50	3.42	<0.001				−12	40	12	Left anterior cingulate (BA 32)
NEWgtOLD: FU > BL, MCI > HC	4.08	3.96	<0.001	<0.001	467	**0.004/0.003**	−10	22	0	Left caudate (head)
NEWgtOLD: FU > BL, MCI > HC	3.93	3.82	<0.001				−4	10	−12	Left anterior cingulate (BA 25)
NEWgtOLD: FU > BL, MCI > HC	3.45	3.38	<0.001				−16	16	10	Left caudate (body)
NEWgtOLD: FU > BL, MCI > HC	4.03	3.91	<0.001	<0.001	395	**0.012/0.005**	2	−76	−38	Right cerebellum – pyramis
NEWgtOLD: FU > BL, MCI > HC	3.88	3.78	<0.001				4	−68	−56	Right cerebellum – inferior semi-lunar
NEWgtOLD: FU > BL, MCI > HC	3.75	3.65	<0.001				4	−76	−54	Right cerebellum – inferior semi-lunar
NEWgtOLD: FU > BL, MCI > HC	3.95	3.85	<0.001	0.005	165	0.57/0.12	46	−42	56	Right inferior parietal lobule (BA 40)
NEWgtOLD: FU > BL, MCI > HC	3.30	3.23	0.001				54	−32	60	Right postcentral gyrus (BA 2)
NEWgtOLD: FU > BL, MCI > HC	3.72	3.63	<0.001	0.001	240	**0.17/0.03**	−14	64	4	Left medial frontal gyrus (BA 10)
NEWgtOLD: FU > BL, MCI > HC	3.13	3.07	0.001				−22	60	26	Left middle frontal gyrus (BA 10)
NEWgtOLD: FU > BL, MCI > HC	3.64	3.56	<0.001	0.007	148	0.70/0.15	−44	−24	−12	Left superior temporal gyrus (BA 22)
NEWgtOLD: FU > BL, MCI > HC	3.29	3.22	0.001				−36	−18	−8	Left claustrum
NEWgtOLD: FU > BL, MCI > HC	3.56	3.48	<0.001	<0.001	385	**0.14/0.005**	−46	−40	22	Left insula (BA 13)
NEWgtOLD: FU > BL, MCI > HC	3.24	3.17	0.001				−36	−54	22	Left superior temporal gyrus (BA 22)
NEWgtOLD: FU > BL, MCI > HC	3.23	3.17	0.001				−30	−52	−6	Left parahippocampal gyrus (BA 19)
NEWgtOLD: FU > BL, MCI > HC	3.51	3.43	<0.001	<0.001	347	**0.27/0.008**	−46	8	14	Left precentral gyrus (BA 44)
NEWgtOLD: FU > BL, MCI > HC	3.42	3.35	<0.001				−46	2	30	Left inferior frontal gyrus (BA 9)
NEWgtOLD: FU > BL, MCI > HC	3.15	3.09	0.001				−42	0	10	Left insula (BA 13)
NEWgtOLD: FU > BL, MCI > HC	3.48	3.40	<0.001	0.013	121	0.90/0.23	38	−46	−12	Right parahippocampal gyrus (BA 19)
NEWgtOLD: FU > BL, MCI > HC	3.47	3.39	<0.001	0.014	119	0.90/0.23	56	14	40	Right middle frontal gyrus (BA 9)
NEWgtOLD: FU > BL, MCI > HC	3.32	3.26	0.001				58	26	28	Right middle frontal gyrus (BA 46)
NEWgtOLD: FU > BL, MCI > HC	3.41	3.34	<0.001	0.001	258	**0.12/0.02**	38	−88	26	Right superior occipital gyrus (BA 19)
NEWgtOLD: FU > BL, MCI > HC	3.30	3.23	0.001				40	−78	10	Right middle temporal gyrus (BA 39)
NEWgtOLD: FU > BL, MCI > HC	3.23	3.17	0.001				24	−90	28	Right cuneus (BA 19)
NEWgtOLD: FU > BL, MCI > HC	3.39	3.32	<0.001	0.020	104	0.97/0.32	6	−28	26	Right cingulate gyrus (BA 23)
NEWgtOLD: FU > BL, MCI > HC	3.28	3.21	0.001	0.006	155	0.65/0.14	−8	−46	64	Left paracentral lobule (BA 5)
NEWgtOLD: FU > BL, MCI > HC	3.16	3.11	0.001	0.021	101	0.98/0.32	−24	28	32	Left middle frontal gyrus (BA 9)
NEWgtOLD: FU > BL, MCI > HC	2.98	2.93	0.002	0.010	132	0.83/0.20	−10	20	60	Left superior frontal gyrus (BA 6)

BOLD, significant at a cluster-wise threshold of p < 0.05 corrected for multiple comparisons (FWE and/or FDR).

BA, Brodmann Area; BL, baseline scan; FU, follow-up scan; HC, healthy control; FWE, family-wise error correction for multiple comparisons; FDR, false discovery rate correction for multiple comparisons; k, number of voxels in cluster; MCI, mild cognitive impairment; MNI, Montreal Neurologic Institute; unc., uncorrected for multiple comparisons.
